# Editorial: Antimicrobial resistance in pediatric infectious diseases: antimicrobial resistance, resistance mechanisms and antimicrobial use

**DOI:** 10.3389/fcimb.2023.1287051

**Published:** 2023-09-26

**Authors:** Dingle Yu, Yuejie Zheng, Adong Shen, Fann Wu, Alexander V. Dmitriev, Mogens Kilian, Yonghong Yang

**Affiliations:** ^1^ Department of Respiratory Medicine, Shenzhen Children’s Hospital, Shenzhen, China; ^2^ Beijing Key Laboratory of Pediatric Respiratory Infection Diseases, Key Laboratory of Major Diseases in Children, Ministry of Education, National Clinical Research Center for Respiratory Diseases, Laboratory of Respiratory Diseases, Beijing Pediatric Research Institute, Beijing Children’s Hospital, Capital Medical University, National Center for Children’s Health, Beijing, China; ^3^ Department of Pathology & Cell Biology, Columbia University Irving Medical Center, New York, NY, United States; ^4^ Institute of Experimental Medicine, Academy of Medical Sciences, Saint Petersburg, Russia; ^5^ Bacterial Population Genetics, Copenhagen University, Copenhagen, Denmark; ^6^ Microbiology Laboratory, National Center for Children’s Health, Beijing Pediatric Research Institute, Beijing Children’s Hospital, Capital Medical University, Beijing, China

**Keywords:** infectious disease, antimicrobial resistance, mechanism, child, antimicrobial use

Antimicrobial Resistance (AMR) occurs when bacteria, viruses, fungi and parasites no longer respond to antimicrobial agents. As a result of drug resistance, antibiotics (usually used for bacteria) and other antimicrobial agents become ineffective and infections become difficult or impossible to treat, increasing the risk of disease spread, severe illness and death. AMR is a global concerning problem, especially in pediatrics. AMR is a serious threat to public health worldwide. Between 2020-2030, patients with antimicrobial resistant infections in Western Pacific region will spend 172 million extra days in hospital, and an estimated 5.2 million deaths will be caused by drug-resistant bacterial infections in the region (WHO, 2023). Children are major consumers of antimicrobial agents and have high rates of AMR. Children’s underdeveloped immune systems make them more susceptible to infectious diseases such as pneumonia and meningitis and are treated with antibiotics. Insufficient understanding of the resistance mechanisms of common pediatric pathogens and lack of pediatric-specific data have both contributed to the overuse and misuse of antibiotics, making antibiotic resistance in pediatric infections a growing threat to public health. Macrolide- and clindamycin-resistant *Streptococcus pneumoniae* and *Bordetella pertussis* are serious problems for children in some countries, such as China. The detection rate of carbapenem-resistant *Enterobacteriaceae* was also higher in children than in adults. Because children are in a special period of growth and development, their pharmacokinetic (PK) and pharmacodynamic (PD) characteristics vary widely, making it difficult to determine age-dependent doses. The lack of pediatric-specific data is also an important cause of the irrational use of antibiotics in children, leading to treatment failure and antibiotic resistance.

This research topic focuses on the antimicrobial-resistant pathogens associated with pediatric patients including *Enterobacteriaceae*, β-hemolytic *Streptococcus* species (*Streptococcus pyogenes*, *Streptococcus agalactiae*, *Streptococcus dysgalactiae subsp. equisimilis*, etc), *Streptococcus pneumoniae*, *Staphylococcus*, *Bordetella pertussis*, *Mycoplasma pneumoniae* and *Mycobacterium tuberculosis*. A better understanding of the resistance phenotype, transfer, and mechanism of antibiotic-resistant bacteria can fill gaps and expand our knowledge of resistant bacterial epidemiology while uncovering interesting patterns of distribution of strain types. Strategies in the battle against antibiotic-resistant bacteria should be compiled. In addition, a understanding of the current situation of most commonly prescribed pediatric medications can lead to create new guidelines to improve antibiotic stewardship. Antibiotic prescribing patterns for children including hospitalized children, outpatients, or children admitted to the emergency department and General Practice, the multi-center survey will be better. In addition, the search for natural antibacterial compounds and chemical synthesis of novel antibacterial products will help to influence antibiotic resistant bacteria. Finally, the study of COVID-19 associated antibiotic resistant bacterial pathogens circulating in Intensive Care Units can reduce the severity of morbidity and mortality caused by COVID-19.

We seek papers on, but not limited to, the following topics:—Antibiotic resistances and multidrug resistances in pediatric pathogens, including resistance phenotypes and genotypes, antibiotic resistance genetic determinants, antibiotic resistance gene transfer.—Antibiotic resistance epidemiology in children.—PK and PD studies of antibiotics in children.—The rationale for use of antibiotics in children.—Novel antibacterial substances for pediatric use. The result of this call is a relatively comprehensive Research Topic of 17 articles regarding such aspects.

## Antibiotic resistances and multidrug resistances

Li et al. give a comprehensive review of the severe problem of macrolide resistance to common pathogens in China. Various common pathogens have shown high resistance rates and high resistance level to macrolides in Chinese children. Yu et al. give a review of penicillin binding protein and *S. pyogenes* (group A *Streptococcus*, GAS) with reduced β-lactam susceptibility. They summarize the current published data on GAS penicillin binding proteins and β-lactam susceptibility, to explore the relationship between them, and to be alert to the emergence of GAS with reduced susceptibility to β-lactams. Cao et al. present a case of spinal muscular atrophy with extensively drug-resistant *Acinetobacter baumannii* pneumonia treated with nebulization combined with intravenous polymyxin B. They present their experience in the diagnosis and treatment of this case and review it in the context of the literature. Wang et al. provide an experimental study about a novel mechanisms of macrolide resistance revealed by *in vitro* selection and genome analysis in *Mycoplasma pneumoniae*. This study presented the first *in vitro* data of induced midecamycin resistance in *M. pneumoniae* and the potential advantage of using midecamycin as an alternative first treatment choice for *M. pneumoniae* infections in patients. Zhuang et al. review the trends and challenges of multi-drug resistance in childhood tuberculosis. This review provides an overview of the current epidemiology of childhood tuberculosis (TB) and drug-resistant tuberculosis (DR-TB), including prevalence, incidence, and mortality. They highlight the urgent need for improved diagnosis and treatment of DR-TB in children.

## Antibiotic resistance epidemiology

Guo et al. provide a dynamic change of serotype distribution and antimicrobial resistance of pneumococcal isolates since PCV13 administration and COVID-19 control in Urumqi, China. Su et al. describe the 10-year trends in multicenter investigation in China about the antibiotic susceptibility and clonal distribution of *Staphylococcus aureus* from pediatric skin and soft tissue infections. Wu et al. present an antimicrobial resistance profile of methicillin-resistant *S. aureus* isolates in children reported from the Infectious Disease Surveillance of Pediatrics (ISPED) surveillance of bacterial resistance, 2016–2021. Wang et al. present the characterization of resistance genes and plasmids from sick children caused by *Salmonella enterica* resistance to azithromycin in Shenzhen, China. Kawata et al. describe the fecal carriage rate of extended-spectrum β-lactamase–producing or carbapenem-resistant *Enterobacterales* among Japanese infants in the community at the 4-month health examination in a rural city. Xiao et al. present the antibiotic susceptibility of *Escherichia coli* isolated from neonates admitted to neonatal intensive care units across China from 2015 to 2020. Jiang et al. discuss the clinical significance of macrolide resistance in pediatric *Mycoplasma pneumoniae* infection during COVID-19 pandemic. Weidmann et al. report the assessing respiratory viral exclusion and affinity interactions through coinfection incidence in a pediatric population during the 2022 resurgence of influenza and RSV.

## PK and PD studies of antibiotics

Peng et al. demonstrate that the vancomycin dosages of 40-60 mg/kg/d are effective and have no vancomycin-related nephrotoxicity adverse effects in children with Gram positive bacterial sepsis. Vancomycin trough concentrations >15 mg/L are not an essential target for these Gram-positive bacterial sepsis patients.

## The rational use of antibiotics

Antibiotics are a double-edged sword, and when used, we must avoid not only overuse, but also underuse. In recent years, countries around the world have introduced antibiotic action plans to control antibiotic resistance in bacteria and strictly control the clinical use of antibiotics, which has led to the underuse of clinical antibiotics (Hsia et al., 2019; Zhang et al., 2019; WHO, 2023). Yu et al. give a case report about rational use of antibiotics, in which 3 cases of septic arthritis in children caused by *S. pyogenes* were reported. Once *S. pyogenes* infection is confirmed, β-lactam antibiotics provide effective treatment, avoiding use of broad-spectrum antibiotics.

Underuse is common, as shown by: 1. not using it in time when it should be used; 2. insufficient dosage; 3. insufficient duration. These lead to an increase in bacterial infections and even epidemics. This phenomenon can be confirmed by the outbreak of GAS epidemic in the UK in 2022 (The Lancet Microbe, 2023; Venkatesan, 2023). Based on the great controversy in the implementation of β-lactam antibiotic skin tests, especially the controversial cephalosporin skin tests in pediatrics, the mechanism and reasons of anaphylaxis to β-lactam antibiotics, the significance of β-lactam antibiotic skin tests, the current state of β-lactam antibiotic skin tests at home and abroad, and the problems of domestic and international skin tests were analyzed to determine a unified standard of β-lactam antibiotic skin tests in pediatrics to prevent and decrease adverse drug reactions, avoid waste of drugs, and a large amount of manpower and material resource consumption. Gao et al. review the state and consideration for skin test of β-lactam antibiotics in pediatrics.

## Novel antibacterial substances

Multi-drug resistant TB (MDR-TB) is often undiagnosed in children due to lack of awareness or under-diagnosis, and the target for children’s DR-TB treatment has only been met in 15% of goals. However, due to age and weight differences, adults and children require different dosages. New medications such as bedaquiline and delamanid have been approved for treating DR-TB. Zhu et al. give a comprehensive review of the advances of new drugs bedaquiline and delamanid in the treatment of multi-drug resistant tuberculosis in children. Their review highlights the use of bedaquiline and delamanid as potential treatments for children with MDR-TB. They summarize their development history, efficacy, safety and potential adverse effects. Further research is necessary to determine the optimal use of these drugs for treating MDR-TB in children.

## Concluding remarks

The problem of antimicrobial resistance in children cannot be ignored. We sincerely thank all contributors and reviewers for their support in putting this timely Research Topic together and hope that the readers will find useful answers to their questions.

## Author contributions

DY: Writing – original draft, Writing – review & editing. YZ: Funding acquisition, Writing – review & editing. AS: Writing – review & editing. FW: Writing – review & editing. AD: Writing – review & editing. MK: Writing – review & editing. YY: Funding acquisition, Resources, Writing – review & editing.

## References

[B1] Hsia Y. Lee B. R. Versporten A. Yang Y. Bielicki J. Jackson C.. (2019). Use of the WHO Access, Watch, and Reserve classification to define patterns of hospital antibiotic use (AWaRe): an analysis of paediatric survey data from 56 countries. Lancet Glob. Health 7 (7), e861–e871. doi: 10.1016/s2214-109x(19)30071-3 31200888

[B2] The Lancet Microbe. (2023). Strep A treatment, working for now. Lancet Microbe 4 (1), e1. doi: 10.1016/S2666-5247(22)00360-3 36565711

[B3] Venkatesan P. (2023). Rise in group A streptococcal infections in England. Lancet Resp. Med. 11 (2), e16. doi: 10.1016/S2213-2600(22)00507-0 36549319

[B4] WHO (2023). Antimicrobial resistance. Available at: https://www.who.int/westernpacific/health-topics/antimicrobial-resistance. (Accessed 9 June, 2023).

[B5] Zhang J. Zheng Y. Yang Y. (2019). Antibiotic prescription patterns in children and neonates in China. Lancet Glob. Health 7 (11), e1496. doi: 10.1016/s2214-109x(19)30406-1 31607463

